# Structure, Process and Outcomes in Patient Engagement

**DOI:** 10.1093/geroni/igab046.574

**Published:** 2021-12-17

**Authors:** Carol Geary, June Eilers, Cheryl Jernigan, Kim Kimminau

**Affiliations:** 1 University of Nebraska Medical Center, University of Nebraska Medical Center, Nebraska, United States; 2 UNMC, Omaha, Nebraska, United States; 3 Retired, Univeristy of Kansas Medical Center, Kansas, United States; 4 MU, MU, Missouri, United States

## Abstract

Although patient engagement in research is gaining acceptance by researchers and funding bodies, descriptions of implementation options and associated outcomes are limited. In this appreciative inquiry of the 12 institutions involved in the Great Plains Collaborative of the Patient-Centered Outcomes Research Network (PCORnet), we interviewed patient engagement officers and patient partners to enhance understanding of approaches to organizational structure, research engagement processes, and associated outcomes of the engaged research. Multiple structures have been identified including operational affiliations in both hospital and multiple university departments. Professional affiliations of patient engagement officers vary widely, including nurses, social workers, and public health professionals, among others. Patient engagement processes also vary, but with the majority using forms of advisory boards. All sites reported outcomes of their work including completed and/or ongoing research and co-authored publications.

